# Unraveling the influence of TTF-1 expression on immunotherapy outcomes in PD-L1-high non-squamous NSCLC: a retrospective multicenter study

**DOI:** 10.3389/fimmu.2024.1399889

**Published:** 2024-07-15

**Authors:** Naoya Nishioka, Hayato Kawachi, Tadaaki Yamada, Motohiro Tamiya, Yoshiki Negi, Yasuhiro Goto, Akira Nakao, Shinsuke Shiotsu, Keiko Tanimura, Takayuki Takeda, Asuka Okada, Taishi Harada, Koji Date, Yusuke Chihara, Isao Hasegawa, Nobuyo Tamiya, Taiki Masui, Natsuki Sai, Masaki Ishida, Yuki Katayama, Kenji Morimoto, Masahiro Iwasaku, Shinsaku Tokuda, Takashi Kijima, Koichi Takayama

**Affiliations:** ^1^ Department of Pulmonary Medicine, Graduate School of Medical Science, Kyoto Prefectural University of Medicine, Kyoto, Kyoto, Japan; ^2^ Department of Thoracic Oncology, Osaka International Cancer Institute, Osaka, Osaka, Japan; ^3^ Department of Respiratory Medicine and Hematology, School of Medicine, Hyogo Medical University, Nishinomiya, Hyogo, Japan; ^4^ Department of Respiratory Medicine, Fujita Health University School of Medicine, Toyoake, Aichi, Japan; ^5^ Department of Respiratory Medicine, Fukuoka University Hospital, Fukuoka, Japan; ^6^ Department of Respiratory Medicine, Japanese Red Cross Kyoto Daiichi Hospital, Kyoto, Kyoto, Japan; ^7^ Department of Respiratory Medicine, Japanese Red Cross Kyoto Daini Hospital, Kyoto, Kyoto, Japan; ^8^ Department of Respiratory Medicine, Saiseikai Suita Hospital, Suita, Osaka, Japan; ^9^ Department of Medical Oncology, Fukuchiyama City Hospital, Fukuchiyama, Kyoto, Japan; ^10^ Department of Pulmonary Medicine, Kyoto Chubu Medical Center, Nantan, Kyoto, Japan; ^11^ Department of Respiratory Medicine, Uji-Tokushukai Medical Center, Uji, Kyoto, Japan; ^12^ Department of Respiratory Medicine, Saiseikai Shigaken Hospital, Ritto, Shiga, Japan; ^13^ Department of Respiratory Medicine, Rakuwakai Otowa Hospital, Kyoto, Kyoto, Japan

**Keywords:** TTF-1, immunotherapy, PD-L1, non-squamous non-small cell lung cancer, antitumor efficacy

## Abstract

**Introduction:**

Several studies explored the association between thyroid transcription factor-1 (TTF-1) and the therapeutic efficacy of immunotherapy. However, the effect of TTF-1 on the therapeutic efficacy of programmed death-1 (PD-1) inhibitor/chemoimmunotherapy in patients with non-squamous non-small cell lung cancer (non-Sq NSCLC) with a programmed death-ligand 1 (PD-L1) tumor proportion score of 50% or more who are highly susceptible to immunotherapy remains unresolved. Therefore, we evaluated whether TTF-1 has a clinical impact on this population.

**Methods:**

Patients with non-Sq NSCLC and high PD-L1 expression who received PD-1 inhibitor monotherapy or chemoimmunotherapy between May 2017 and December 2020 were retrospectively enrolled. Treatment efficacy was compared after adjusting for baseline differences using propensity score matching.

**Results:**

Among the 446 patients with NSCLC with high PD-L1 expression, 266 patients with non-Sq NSCLC were analyzed. No significant differences in therapeutic efficacy were observed between the TTF-1-positive and -negative groups in the overall and propensity score-matched populations. Of chemoimmunotherapy, pemetrexed containing regimen significantly prolonged progression-free survival compared to chemoimmunotherapy without pemetrexed, regardless of TTF-1 expression (TTF1 positive; HR: 0.46 (95% Confidence interval: 0.26-0.81), p<0.01, TTF-1 negative; HR: 0.29 (95% Confidence interval: 0.09-0.93), p=0.02).

**Discussion:**

TTF-1 expression did not affect the efficacy of PD-1 inhibitor monotherapy or chemoimmunotherapy in patients with non-Sq NSCLC with high PD-L1 expression. In this population, pemetrexed-containing chemoimmunotherapy demonstrated superior anti-tumor efficacy, irrespective of TTF-1 expression.

## 1 Introduction

The standard of treatment for non-small cell lung cancer (NSCLC) patients, excluding those with driver mutations, involves the use of immune checkpoint inhibitors. Especially for individuals with high programmed death-ligand 1 (PD-L1) expression, PD-1/PD-L1 inhibitor monotherapy or chemoimmunotherapy is the preferred treatment in most cases. Although both PD-1/PD-L1 inhibitor monotherapy and chemoimmunotherapy are effective first-line treatment regimens for PD-L1 high NSCLC patients, several systematic reviews have suggested that chemoimmunotherapy may be more effective under certain conditions (1, 2). In addition, for non-squamous NSCLC patients, there are also many options for chemoimmunotherapy, including regimens that incorporate pemetrexed and paclitaxel. As a result, determining the optimal treatment for each patient is challenging, and currently, there is no clear distinction between these options.

Thyroid transcription factor-1 (TTF-1), known as a lineage-specific transcription factor, is a gene-regulating protein found in the thyroid, lungs, and brain (3, 4). In normal lung tissues, it is expressed in type II alveolar epithelial and Clara cells and promotes the transcription of surfactant protein genes (5). TTF-1 has been reported to be associated with lung cancer development; hence, TTF-1 is expressed in 60–80% of the cases of non-squamous non-small cell lung cancer (non-Sq NSCLC) and is commonly used for the histological diagnosis of lung cancer (6).

TTF-1 expression has been reported as a potential prognostic factor in patients with NSCLC and has also been previously reported to be significantly associated with therapeutic response to cytotoxic anticancer agents, particularly pemetrexed, with favorable results in TTF-1 positive patients (7, 8). Similarly, several reports on immunotherapy alone or chemoimmunotherapy have indicated that TTF-1-positive non-squamous NSCLC patients tend to have a better therapeutic response (9–11). However, there has been no evaluation of the impact of TTF-1 expression on the therapeutic effect of immunotherapy alone or chemotherapy in patients with NSCLC with high PD-L1 expression who are strongly affected by immunotherapy. Therefore, we evaluated whether TTF-1 has a clinical impact on this population.

## 2 Methods

### 2.1 Patients and study designs

We conducted a retrospective, multicenter cohort study of patients with NSCLC who received at least one dose of a programmed death-1 (PD-1)/PD-L1 inhibitor, with or without chemotherapy, from May 2017 to December 2020. This study was approved by the ethics review board of Kyoto Prefectural University of Medicine and was conducted with the consent of the ethics review boards of the 13 hospitals involved in the study. The inclusion criteria were as follows: (1) histologically or cytologically diagnosed NSCLC (2) histologically proven stage III–IV according to TNM staging (AJCC eighth edition) or postoperative recurrence (3) PD-L1 tumor proportion score ≥ 50%. The exclusion criteria were as follows: (1) neuroendocrine carcinoma or squamous cell carcinoma and (2) not evaluated for TTF-1 expression in the tumor. All clinical data were collected from electronic medical records and PD-L1 scores were analyzed using PD-L1 immunohistochemistry with a 22C3 pharmDx antibody (clone 22C3; Dako North America, Inc.). TTF-1 was analyzed using mouse monoclonal anti-TTF-1 antibody clone 8G7G3/1, mouse monoclonal anti-TTF-1 antibody clone SPT24, rabbit monoclonal anti-TTF-1 antibody clone SP141. TTF-1 expression was all assessed via immunohistochemistry on lung cancer tissue samples obtained during surgery, bronchoscopy, or CT-guided biopsy at diagnosis in all centers, and pathologists at each facility determined the positivity of the results.

### 2.2 Assessment outcome

We investigated the relationship between TTF-1 expression and the clinical outcomes in patients who received pembrolizumab monotherapy or chemoimmunotherapy. Furthermore, each regimen’s outcomes were evaluated and stratified according to tumor TTF-1 negative and positive patients. When comparing the treatment outcomes between the TTF-1-negative and TTF-1-positive groups, we adjusted for significant differences in baseline characteristics using propensity score matching for the following variables: Eastern Cooperative Oncology Group (ECOG-PS), histology, brain metastasis, and treatment regimen. Matching was performed using nearest-neighbor matching at a 1:1 ratio without replacement with a caliper of 0.2. Progression-free survival (PFS) was measured from the treatment initiation date to the date of tumor progression or death, whichever occurred first, while overall survival (OS) was measured from the date of first-line treatment to the date of any-cause death.

### 2.3 Statistical analysis

Binary variables were analyzed using Fisher’s exact test. PFS and OS were estimated using the Kaplan–Meier method and compared using the log-rank test. The Cox proportional hazards model was used to determine the association between patient characteristics and survival outcomes. All statistical analyses were performed using EZR (Saitama Medical Center, Jichi Medical University, Saitama, Japan), and statistical significance was set at p< 0.05 (12).

## 3 Results

### 3.1 Patient characteristics and treatment

Of the 446 consecutive patients with advanced NSCLC who initially received immune checkpoint inhibitor (ICI) monotherapy or chemoimmunotherapy, 266 patients with non-Sq NSCLC were enrolled in this study (**Supplementary Figure 1**). The exclusion criteria were as follows: (a) the histology of the patients’ tumors was squamous cell carcinoma (n=123) and (b) the evaluation of TTF-1 expression was not conducted (n=57). Patient characteristics are summarized in **Table 1**. The median patient age was 70 years (range:36–90) years. The majority were male (74.1%), had an ECOG-PS of 0–1 (84.6%), and had adenocarcinoma (79.7%). Other histologic types included large cell lung cancer (1.1%), pleomorphic carcinoma (4.1%), sarcomatoid tumors (0.8%), and not otherwise specified (NOS) (14.3%). The PD-L1 tumor proportion score (TPS) was available for all patients: 155 (58.3%), 50–79%, and 111 (41.7%) had 80–100%.

**Table 1:** Baseline characteristics of patients in the propensity score-matched population.

Characteristic	Total n=90	TTF-1 negative n=45	TTF-1 positive n=45	*p*-value
Age, y				
Median (range)	70 [36–90]	69 [36–90]	71 [46–86]	0.15
Gender				
Male Female	68 (75.6) 22 (24.4)	34 (75.6) 11 (24.4)	34 (75.6) 11 (24.4)	1.00
ECOG-PS				
0-1 **≥** 2	78 (86.7) 12 (13.3)	39 (86.7) 6 (13.3)	39 (86.7) 6 (13.3)	1.00
Stage				
IVA IVB Postoperative recurrence	29 (32.2) 48 (53.3) 13 (14.4)	12 (26.7) 23 (51.1) 10 (22.2)	17 (37.7) 25 (55.6) 3 (6.7)	0.14
Histology				
Adeno Others LCNEC Pleomorphic carcinoma Sarcomatoid carcinoma NOS	62 (68.9) 28 (31.1) 2 (2.2) 5 (5.6) 1 (1.1) 20 (22.2)	31 (68.9) 14 (31.1) 1 (2.2) 2 (4.5) 1 (2.2) 10 (22.2)	31 (68.9) 14 (31.1) 1 (2.2) 3 (6.7) 0 10 (22.2)	1.00
Liver metastasis	11 (12.2)	6 (13.3)	5 (11.1)	1.00
Brain metastasis	14 (15.6)	7 (15.6)	7 (15.6)	1.00
Programmed cell death ligand 1 tumor proportion score, %				
50-89 90-100	50 (55.6) 40 (44.4)	25 (55.6) 20 (44.4)	25 (55.6) 20 (44.4)	1.00
Treatment regimen				
Pembrolizumab monotherapy Chemoimmunotherapy Platinum/pemetrexed/pembrolizumab Platinum/nab-paclitaxel/pembrolizumab Carboplatin/paclitaxel/bevacizumab/ atezolizumab Carboplatin/nab-paclitaxel/atezolizumab	49 (62.4) 41 (37.6) 25 (22.9) 7 (5.3) 6 (6.0) 3 (3.4)	27 (60.0) 18 (40.0) 10 (14.1) 3 (6.7) 4 (8.9) 1 (2.2)	22 (48.9) 23 (51.1) 15 (26.2) 4 (8.9) 2 (4.4) 2 (4.4)	0.40

ECOG-PS, Eastern Cooperative Oncology Group performance status; TTF-1, Thyroid transcription factor-1; LCNEC, Large cell neuroendocrine carcinoma; NOS, Not otherwise specified.

We classified patients into TTF-1 positive or negative groups, as shown in **Supplementary Table 1**. Between the two groups, the TTF-1-negative group exhibited a significantly lower rate of PS 0–1, with more than half of the histologic types being non-adenocarcinoma. In contrast, the TTF-1-positive group had a significantly higher brain metastasis incidence. Furthermore, the TTF-1 positive group had a higher percentage of pemetrexed regimens than the TTF-1 negative group.

### 3.2 Treatment outcomes in all patients with TTF-1 positive or negative

**Figure 1:**
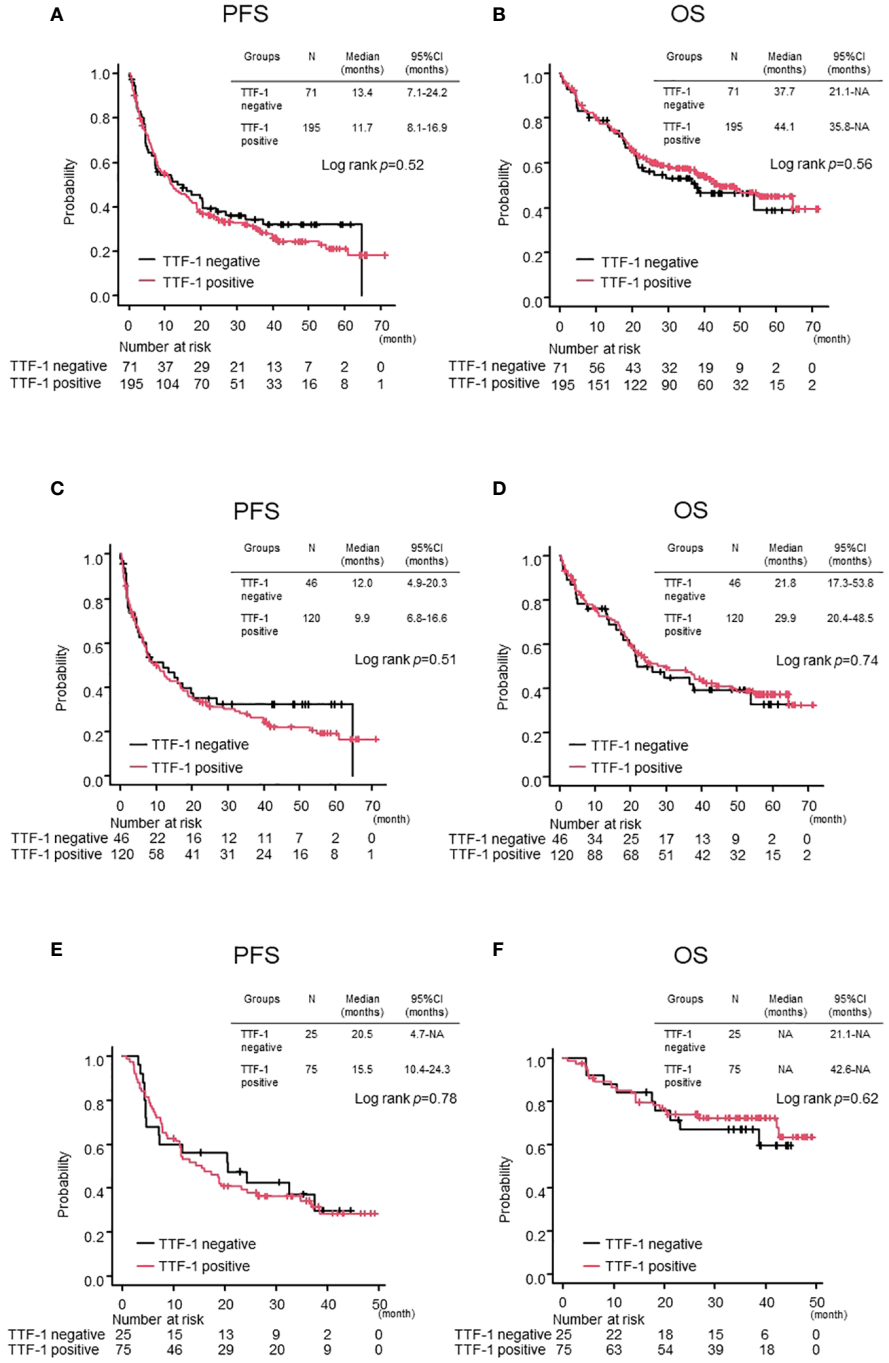
Comparison of treatment efficacy between TTF-1 positive and negative groups in the overall population. Kaplan–Meier curves for **(A)** progression-free survival (PFS) and **(B)** overall survival (OS) in the entire population. PFS **(C, D)** OS in the Pembrolizumab monotherapy population and PFS **(E, F)** OS in the Chemoimmunotherapy population.

As of the cutoff date on May 15, 2023, with a median follow-up of 42.6 months, 189 out of 266 patients (71%) had experienced progressive disease. In the overall population, there were no significant differences between TTF-1 positive and negative groups in terms of PFS and OS (TTF-1 positive vs TTF-1 negative; median PFS 11.7 months vs 13.4 months p=0.52, median OS 44.1 months vs 37.7 months p=0.56) (**Figures 1A, B**). Additionally, after dividing the entire population into pembrolizumab monotherapy and chemoimmunotherapy groups, no significant differences were observed in either PFS or OS between the TTF-1 positive and TTF-1 negative subgroups within either group (**Figures 1C–F**). Thus, the expression level of TTF1 did not significantly affect the efficacy of pembrolizumab monotherapy or chemoimmunotherapy in PD-L1 high-expressing patients with non-Sq NSCLC.

### 3.3 Patient characteristics and treatment in the propensity score-matched population

The baseline covariates included in the propensity score calculation were the ECOG-PS score, histology, brain metastasis, and treatment regimen. After adjusting for propensity score matching, there were 45 patients in each of the TTF-1 positive and negative groups (**Table 1**). As a result, all categories showed no obvious statistically significant differences. Regarding treatment, among those in the TTF-1-positive group, 22 patients received ICI monotherapy and 23 received chemoimmunotherapy. In the TTF-1-negative group, 27 patients received ICI monotherapy, and 18 patients received chemoimmunotherapy.

### 3.4 Treatment outcomes in the propensity score-matched population

**Figure 2:**
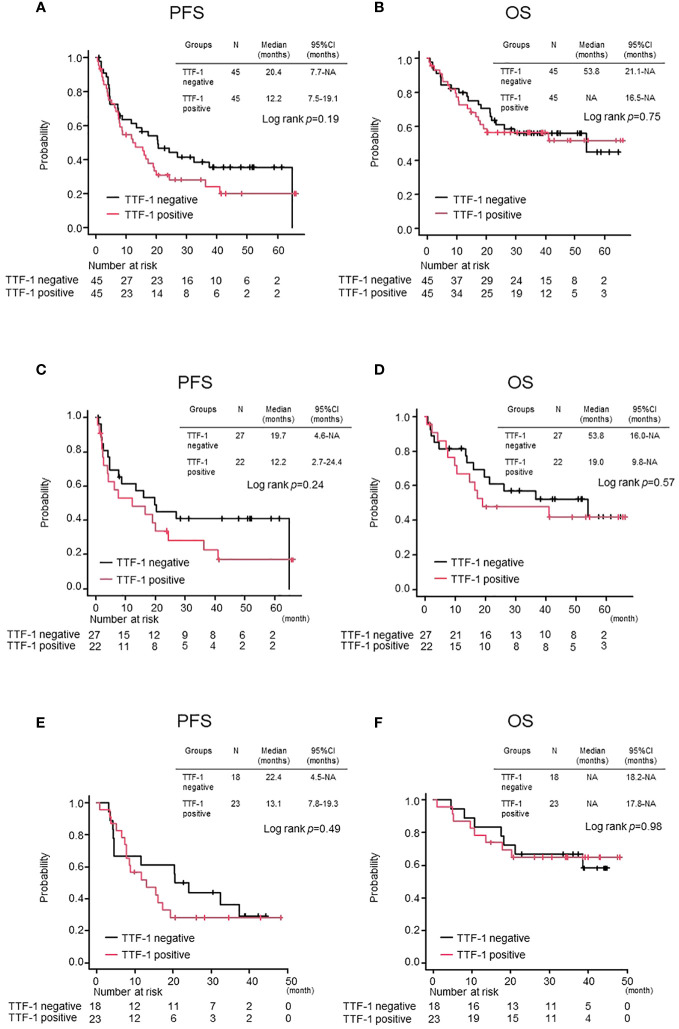
Comparison of treatment efficacy between TTF-1 positive and negative groups after propensity matching. Kaplan–Meier curves for **(A)** progression-free survival (PFS) and **(B)** overall survival (OS) in the entire population. PFS **(C, D)** OS in the Pembrolizumab monotherapy population and PFS **(E, F)** OS in the Chemoimmunotherapy population.

There was no statistically significant difference in either median OS or median PFS between the TTF-1 positive and negative groups (**Figures 2A, B**). Similarly, when these populations were evaluated separately in the ICI monotherapy and chemoimmunotherapy cohorts, no significant differences in the median PFS and OS were observed (**Figures 2C–F**). Based on these observations, despite incorporation into the propensity score-matched analysis, the TTF1 expression level did not significantly affect the effectiveness of pembrolizumab monotherapy or chemoimmunotherapy in patients with PD-L1 high-expressing non-Sq NSCLC.

### 3.5 Comparative analysis of treatment efficacy across different treatment regimens stratified by TTF-1 expression

**Figure 3:**
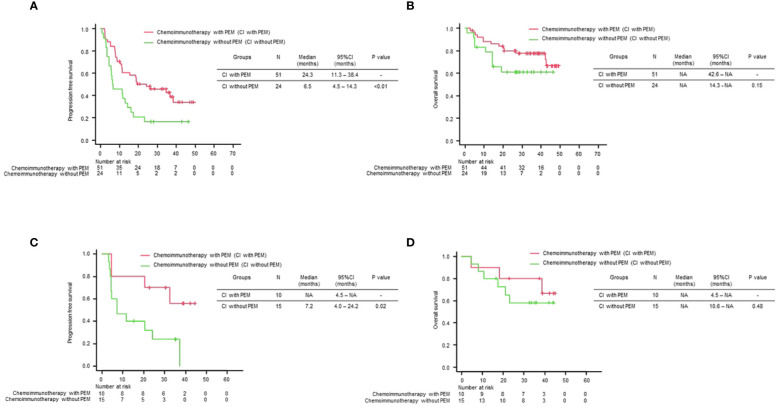
Comparison of treatment efficacy across different treatment regimens stratified by TTF-1 Expression. Kaplan–Meier curves of **(A)** progression-free survival (PFS) and **(B)** overall survival (OS) for each treatment regimen in the TTF-1 positive non-squamous NSCLC population, and Kaplan–Meier curves of **(C)** PFS and **(D)** OS for each treatment regimen in the TTF-1 negative non-squamous NSCLC population.

Next, we compared the PFS and OS of the chemoimmunotherapy population with positive/negative TTF-1 expression in two groups: chemoimmunotherapy with pemetrexed (CI with PEM) and chemoimmunotherapy without pemetrexed (CI without PEM). Baseline characteristics of patients receiving chemoimmunotherapy stratified by TTF-1 expression are shown in **Supplementary Table 2**. Among the subset of the TTF-1 positive group, CI with PEM demonstrated a statistically significant PFS prolongation compared to CI without PEM (CI with PEM vs CI without PEM: HR: 0.46 (95% Confidence interval: 0.26-0.81), p<0.01, **Figure 3A**). Subsequently, in terms of OS, the CI with PEM group showed a tendency to extend compared to the CI without PEM group, regardless of statistical significance (CI with PEM vs. CI without PEM: HR: 0.54 (95% Confidence interval: 0.23-1.26), p=0.15, **Figure 3B**). Among the subset of the TTF-1 negative group, CI with PEM demonstrated a prolonged PFS compared to CI without PEM (CI with PEM vs. CI without PEM: HR: 0.29 (95% Confidence interval: 0.09-0.93), p=0.02, **Figure 3C**). However, given the immaturity of the OS data, there was no significant difference (CI with PEM vs. CI without PEM: HR: 0.60 (95% Confidence interval: 0.15-2.46), p=0.48, **Figure 3D**).

These results indicate that pemetrexed-containing chemoimmunotherapy potentially improves clinical outcomes in patients with PD-L1 high-expressing non-Sq NSCLC, regardless of the TTF1 expression level, compared with non-pemetrexed chemoimmunotherapy.

## 4 Discussion

This study is one of the largest studies to explore the impact of TTF-1 expression in patients with NSCLC and high PD-L1 expression who have undergone immunotherapy. In this cohort, there were no significant differences in PFS and OS between TTF-1-positive and negative populations. Additionally, we assessed the treatment regimen efficacy and stratified TTF-1 expression (positive/negative). Our findings revealed that chemoimmunotherapy with pemetrexed showed a tendency toward prolonged PFS compared with other regimens, irrespective of TTF-1 expression. Regarding OS, TTF-1-positive patients who received chemotherapy with pemetrexed had prolonged OS compared to the other groups. However, no significant difference in OS was observed in TTF-1-negative patients.

In TTF-1-positive NSCLC, especially in TTF-1-positive lung adenocarcinomas, high differentiation is often observed. Conversely, TTF-1-negative NSCLC cells exhibit lower differentiation and coexist with squamous cell carcinomas derived from atypical mucous column epithelial cells (13). Indeed, in this study, the TTF-1-positive group exhibited a significantly higher proportion of adenocarcinomas and a smaller proportion of non-adenocarcinoma NSCLCs than the TTF-1-negative group. Additionally, non-squamous and non-adeno NSCLCs are associated with a poorer prognosis than adenocarcinoma, suggesting that TTF-1 negative NSCLC has a biologically inferior prognosis compared to TTF-1 positive NSCLC (14). In terms of response to systemic chemotherapy, several previous studies have also indicated that TTF-1-negative NSCLC exhibits a less favorable therapeutic response with pemetrexed-based chemotherapy than TTF-1-positive NSCLC (7, 15, 16). Recent studies have reported that this result also holds true for chemoimmunotherapy (9, 10, 17–20). Actually, our previous study similarly reported a significantly lower therapeutic response to chemoimmunotherapy in the TTF-1-negative population compared to the TTF-1-positive population (11). However, this study, which demonstrated that TTF-1 expression does not affect the therapeutic efficacy of immunotherapy and chemoimmunotherapy, contrasts with the outcomes of previous studies. One hypothesis that could explain these contradictory results is the relationship between TTF-1 and PD-L1 expression levels in advanced NSCLC. In our previous study, we reported that PD-L1 expression was significantly higher in the TTF-1-positive population than in the TTF-1-negative population (11). This association between PD-L1 and TTF-1 expression levels has been reported in several other studies with the same trend (21, 22). It is therefore possible that one of the reasons for the low response to ICI treatment in the overall TTF-1-negative population was influenced by the relatively low PD-L1 expression. Consequently, it is possible that in this study, which was limited to PD-L1-high expressing patients with high efficacy of immunotherapy, there was no difference in response to ICI monotherapy or chemoimmunotherapy, regardless of TTF-1 status.

In this study, regardless of TTF-1 expression, chemoimmunotherapy with pemetrexed tended to prolong PFS compared to chemoimmunotherapy without pemetrexed. This result contradicts previous reports suggesting that conventional pemetrexed-based chemotherapy without immunotherapy results in shorter PFS and OS in TTF-1-negative populations. Several factors may have contributed to this discrepancy. First, there may be differences in the anti-tumor efficacies of immune checkpoint inhibitors. In this study, pemetrexed regimens included platinum + pemetrexed + pembrolizumab, whereas pemetrexed-free chemoimmunotherapy included mostly atezolizumab-based regimens, with the rest being pembrolizumab-based. Multiple immune checkpoint inhibitors, each with different anti-tumor effects, have been reported in clinical trials (23, 24). Second, pemetrexed, a cytotoxic anticancer agent, may have adjunctive effects that enhance the efficacy of immunotherapy. Pemetrexed induces the transcriptional activation of PD-L1 (encoded by CD274) by deactivating thymidylate synthase (TS) in NSCLC cells (25). This in turn activates T lymphocytes when combined with anti-PD-1/PD-L1 therapy. Compared with other anticancer agents, pemetrexed has a more pronounced effect on PD-1/PD-L1 inhibition. Especially in populations more likely to benefit from immunotherapy, such as those with high PD-L1 expression, the adjunctive effect of activating immunotherapy may have a greater impact than the reduced therapeutic effect of pemetrexed owing to TTF-1 negativity. Further large-scale studies are required to confirm these hypotheses.

This study had several limitations. Firstly, this was a retrospective study conducted only in Japan, and various confounding factors may have been involved. Specifically, the comparison of treatment regimens for each TTF-1 expression group, especially for chemoimmunotherapy, involved a few patients, precluding a comprehensive multivariate analysis. Second, despite propensity score matching, unknown factors may have influenced the results, making it challenging to completely align patient backgrounds based on TTF-1 expression.

In patients with NSCLC and high PD-L1 expression, tumor TTF-1 expression did not affect the therapeutic efficacy of PD-1 inhibitor monotherapy or chemoimmunotherapy. Moreover, while previous reports have indicated significant variations in the therapeutic effect of pemetrexed based on TTF-1 expression, the current study suggests that chemoimmunotherapy, including pemetrexed, may exhibit superior therapeutic efficacy compared to other regimens, irrespective of TTF-1 expression.

## Data availability statement

The raw data supporting the conclusions of this article will be made available by the authors, without undue reservation.

## Ethics statement

The studies involving humans were approved by The Ethics Review Board of Kyoto Prefectural University of Medicine. The studies were conducted in accordance with the local legislation and institutional requirements. Written informed consent for participation was not required from the participants or the participants’ legal guardians/next of kin because We implemented an opt-out approach, and therefore, direct informed consent was not conducted. Participants were given the option to decline participation.

## Author contributions

NN: Writing – original draft, Conceptualization, Investigation, Methodology, Software, Validation, Formal analysis. HK: Data curation, Writing – review & editing, Resources. TY: Writing – review & editing, Formal analysis, Investigation, Methodology, Project administration, Validation. MT: Resources, Writing – review & editing. YN: Resources, Writing – review & editing. YG: Resources, Writing – review & editing. AN: Resources, Writing – review & editing. SS: Resources, Writing – review & editing. KKT: Resources, Writing – review & editing. TT: Resources, Writing – review & editing. AO: Resources, Writing – review & editing. TH: Resources, Writing – review & editing. KD: Resources, Writing – review & editing. YC: Resources, Writing – review & editing. IH: Resources, Writing – review & editing. NT: Resources, Writing – review & editing. TM: Resources, Writing – review & editing. NS: Resources, Writing – review & editing. MIs: Resources, Writing – review & editing. YK: Resources, Writing – review & editing. KM: Resources, Writing – review & editing. MIw: Resources, Writing – review & editing. ST: Resources, Writing – review & editing. TK: Resources, Supervision, Writing – review & editing. KCT: Resources, Supervision, Writing – review & editing.
