# Supplementary figures and images for: Unraveling the influence of TTF-1 expression on immunotherapy outcomes in PD-L1-high non-squamous NSCLC: a retrospective multicenter study
Source: Front Immunol. 2024 Jul 15;15:1399889. doi: 10.3389/fimmu.2024.1399889 (PMC11284020; doi:10.3389/fimmu.2024.1399889)

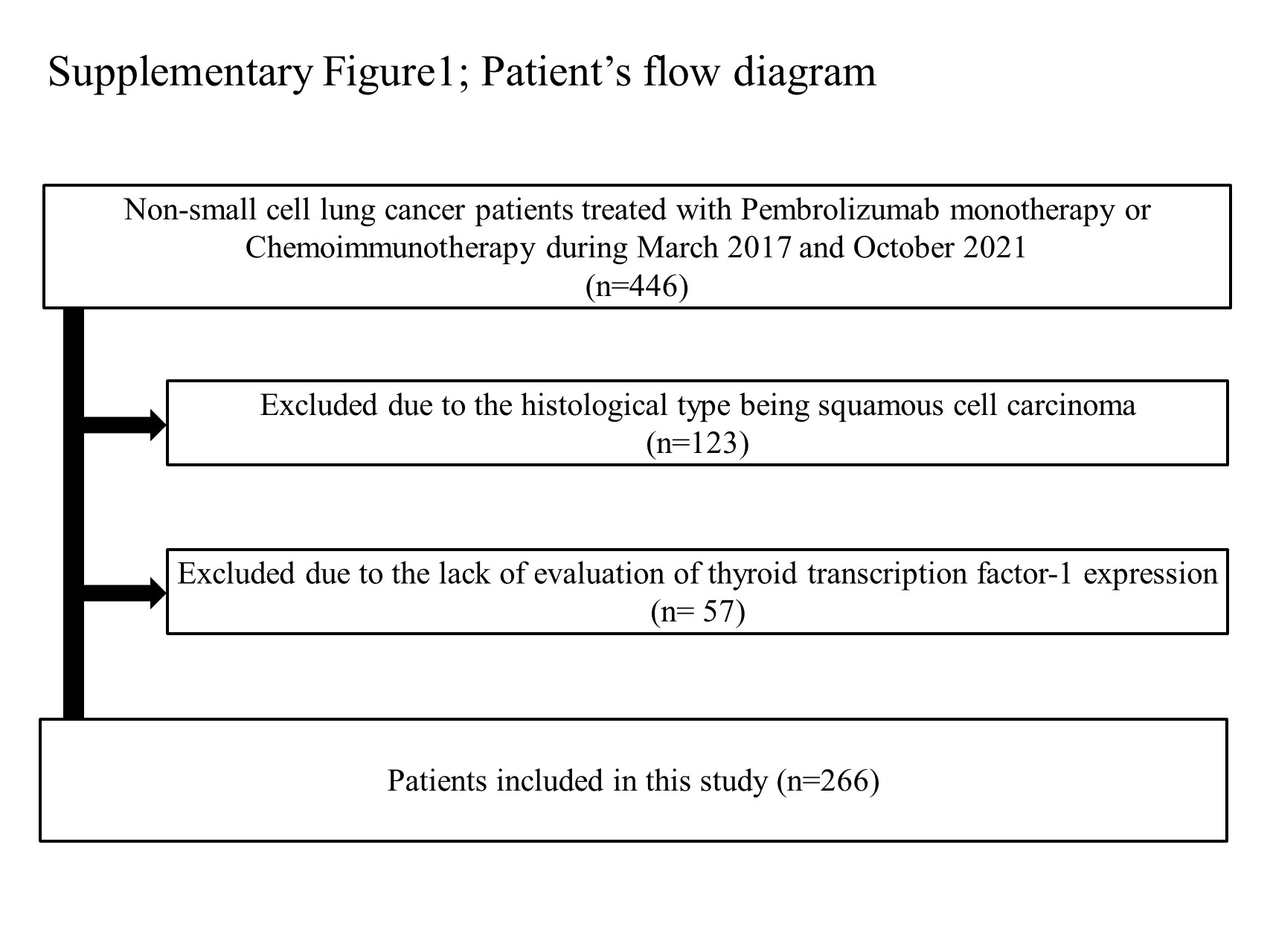

Supplement: Supplementary file 1 [file Image_1.jpeg]
