# Supplementary material for: Unraveling the influence of TTF-1 expression on immunotherapy outcomes in PD-L1-high non-squamous NSCLC: a retrospective multicenter study
Source: Front Immunol. 2024 Jul 15;15:1399889. doi: 10.3389/fimmu.2024.1399889 (PMC11284020; doi:10.3389/fimmu.2024.1399889)
Supplement: Supplementary file 2 [file Table_1.docx]

**Supplementary Table 1. Baseline characteristics of patients included in the study**

| Characteristic | Total  n=266 | TTF-1 negative  n=71 | TTF-1 positive  n=195 | *p*-value |
| --- | --- | --- | --- | --- |
| Age, y  Median (range) | 70 [36–90] | 69 [36–90] | 71 [40–86] | 0.78 |
| Gender  Male  Female | 197 (74.1)  69 (25.9) | 53 (74.6)  18 (25.4) | 144 (73.8)  51 (26.2) | 1.00 |
| ECOG-PS  0-1  **≥** 2 | 225 (84.6)  41 (15.4) | 53 (74.6)  18 (25.4) | 172 (88.2)  23 (11.8) | 0.01 |
| Stage  IVA  IVB  Postoperative recurrence | 84 (31.5)  143 (53.8)  39 (14.7) | 22 (31.0)  37 (52.1)  12 (16.9) | 62 (31.8)  106 (54.4)  27 (13.8) | 0.86 |
| Histology  Adeno  Others  *LCNEC  *Pleomorphic carcinoma  *Sarcomatoid carcinoma  *NOS | 212 (79.7)  54 (20.3)  3 (1.1)  11 (4.1)  2 (0.8)  38 (14.3) | 31 (43.7)  40 (56.3)  2 (2.8)  8 (11.3)  2 (2.8)  28 (39.4) | 181 (92.8)  14 (7.2)  1 (0.5)  3 (1.6)  0  10 (5.1) | <0.01 |
| Liver metastasis | 37 (13.9) | 8 (11.3) | 29 (14.9) | 0.55 |
| Brain metastasis | 60 (22.6) | 10 (14.1) | 50 (25.6) | 0.04 |
| Programmed cell death ligand 1 tumor proportion score, %  50-89  90-100 | 155 (58.3)  111 (41.7) | 39 (54.9)  32 (45.1) | 116 (59.5)  79 (40.5) | 0.57 |
| Treatment regimen  Pembrolizumab monotherapy  Chemoimmunotherapy  　　Platinum/pemetrexed/pembrolizumab  Platinum/nab-paclitaxel/pembrolizumab  　　 Carboplatin/paclitaxel/bevacizumab/atezolizumab  Carboplatin/nab-paclitaxel/atezolizumab | 166 (62.4)  100 (37.6)  61 (22.9)  14 (5.3)  16 (6.0)  9 (3.4) | 46 (64.8)  25 (35.2)  10 (14.1)  8 (11.3)  5 (7.0)  2 (2.8) | 120 (61.5)  75 (38.5)  51 (26.2)  6 (3.1)  11 (5.6)  7 (3.6) | 0.67 |

ECOG-PS, Eastern Cooperative Oncology Group performance status; TTF-1, Thyroid transcription factor-1; LCNEC, Large cell neuroendocrine carcinoma; NOS, Not otherwise specified
