# Supplementary material for: Unraveling the influence of TTF-1 expression on immunotherapy outcomes in PD-L1-high non-squamous NSCLC: a retrospective multicenter study
Source: Front Immunol. 2024 Jul 15;15:1399889. doi: 10.3389/fimmu.2024.1399889 (PMC11284020; doi:10.3389/fimmu.2024.1399889)
Supplement: Supplementary file 3 [file Table_2.docx]

**Supplementary Table 2. Baseline characteristics of patients receiving chemoimmunotherapy stratified**

**by TTF-1 expression: comparison of regimens with and without pemetrexed**

**(A) TTF-1 positive population**

| Characteristic | Total  n=75 | Chemoimmunotherapy | | *p*-value |
| --- | --- | --- | --- | --- |
|  |  | With pemetrexed  n=51 | Without pemetrexed  n=24 |  |
| Age, y  Median (range) | 68 [40–86] | 68 [40-86] | 65 [46–77] | 0.78 |
| Gender  Male  Female | 54 (72.0)  21 (28.0) | 38 (74.5)  13 (25.5) | 16 (66.7)  8 (33.3) | 0.58 |
| ECOG-PS  0-1  **≥** 2 | 71 (94.7)  4 (5.3) | 48 (94.1)  3 (5.9) | 23 (95.8)  1 (4.2) | 1.00 |
| Stage  IVA  IVB  Postoperative recurrence | 17 (22.7)  47 (62.7)  11 (14.7) | 13 (25.5)  32 (62.7)  6 (11.8) | 4 (16.7)  15 (62.5)  5 (20.8) | 0.49 |
| Histology  Adeno  Others  LCNEC  Pleomorphic carcinoma  Sarcomatoid carcinoma  NOS | 68 (90.7)  7 (9.3)  1 (1.3)  1 (1.3)  0  5 (6.7) | 48 (94.1)  3 (5.9)  1 (1.9)  1 (1.9)  0  1 (1.9) | 20 (83.3)  4 (16.7)  0  0  0  4 (16.7) | 0.20 |
| Liver metastasis | 15 (20.0) | 9 (17.6) | 6 (25.0) | 0.54 |
| Brain metastasis | 18 (24.0) | 10 (19.6) | 8 (33.3) | 0.25 |
| Programmed cell death ligand 1 tumor proportion score, %  50-89  90-100 | 52 (69.3)  23 (30.7) | 34 (66.7)  17 (33.3) | 18 (75.0)  6 (25.0) | 0.59 |
| Treatment regimen  Platinum/pemetrexed/pembrolizumab  Platinum/nab-paclitaxel/pembrolizumab Carboplatin/paclitaxel/bevacizumab/atezolizumab  Carboplatin/nab-paclitaxel/atezolizumab | 51 (68.0)  6 (8.0)  11 (14.7)  7 (9.3) | 51 (100)  0  0  0 | 0  6 (25.0)  11 (45.8)  7 (29.2) |  |

ECOG-PS, Eastern Cooperative Oncology Group performance status; TTF-1, Thyroid transcription factor-1; LCNEC, Large cell neuroendocrine carcinoma; NOS, Not otherwise specified

**(B) TTF-1 negative population**

| Characteristic | Total  n=25 | Chemoimmunotherapy | | *p*-value |
| --- | --- | --- | --- | --- |
|  |  | With pemetrexed  n=10 | Without pemetrexed  n=15 |  |
| Age, y  Median (range) | 69 [36–77] | 69 [36-73] | 69 [46–77] | 0.20 |
| Gender  Male  Female | 18 (72.0)  7 (28.0) | 8 (80.0)  2 (20.0) | 10 (66.7)  5 (33.3) | 0.66 |
| ECOG-PS  0-1  **≥** 2 | 22 (88.0)  3 (12.0) | 10 (100)  0 | 12 (80.0)  3 (20.0) | 0.25 |
| Stage  IVA  IVB  Postoperative recurrence | 8 (32.0)  15 (60.0)  2 (8.0) | 1 (10.0)  7 (70.0)  2 (20.0) | 7 (46.7)  8 (53.3)  0 | 0.06 |
| Histology  Adeno  Others  LCNEC  Pleomorphic carcinoma  Sarcomatoid carcinoma  NOS | 13 (52.0)  12 (48.0)  0  2 (8.0)  0  10 (40.0) | 9 (90.0)  1 (10.0)  0  0  0  1 (10.0) | 4 (26.7)  11 (73.3)  0  2 (13.3)  0  9 (60.0) | 0.004 |
| Liver metastasis | 2 (8.0) | 1 (10.0) | 1 (6.7) | 1.00 |
| Brain metastasis | 4 (16.0) | 1 (10.0) | 3 (20.0) | 0.63 |
| Programmed cell death ligand 1 tumor proportion score, %  50-89  90-100 | 13 (52.0)  12 (48.0) | 6 (60.0)  4 (40.0) | 7 (46.7)  8 (53.3) | 0.69 |
| Treatment regimen  Platinum/pemetrexed/pembrolizumab  Platinum/nab-paclitaxel/pembrolizumab Carboplatin/paclitaxel/bevacizumab/atezolizumab  Carboplatin/nab-paclitaxel/atezolizumab | 10 (40.0)  8 (32.0)  5 (20.0)  2 (8.0) | 10 (100)  0  0  0 | 0  8 (53.3)  5 (33.3)  2 (13.3) |  |

ECOG-PS, Eastern Cooperative Oncology Group performance status; TTF-1, Thyroid transcription factor-1; LCNEC, Large cell neuroendocrine carcinoma; NOS, Not otherwise specified
